# Analysis of the safety and efficacy of different plasma concentrations of pirfenidone in patients with idiopathic pulmonary fibrosis

**DOI:** 10.3389/fphar.2022.1055702

**Published:** 2022-11-29

**Authors:** Hui Li, Jing Yang, Shanshan Chen, Peile Wang, Xueqing Yu, Qingwei Zhou, Xiaojian Zhang, Guojun Zhang

**Affiliations:** ^1^ Department of Respiratory Medicine, Henan key Laboratory of Interstitial Lung Diseases and Lung Transplantation Medicine, Henan Respiratory Disease Clinical Medical Research Center, The First Affiliated Hospital of Zhengzhou University, Zhengzhou, China; ^2^ Department of Pharmacy, Henan Engineering Research Center for Application and Translation of Precision Clinical Pharmacy, The First Affiliated Hospital of Zhengzhou University, Zhengzhou University, Zhengzhou, China; ^3^ Department of Respiratory Diseases, The First Affiliated Hospital of Henan University of Chinese Medicine, Zhengzhou, China

**Keywords:** pirfenidone, idiopathic pulmonary fibrosis, plasma concentration, safety, efficacy

## Abstract

The high incidence and mortality of idiopathic pulmonary fibrosis (IPF) have led to the widespread use of antifibrotic drugs such as pirfenidone; however, the associated adverse reactions greatly vary among individuals and the dose is not fixed. To date, no reliable blood concentration range of pirfenidone is available to monitor adverse reactions and clinical efficacy. This real study assessed the efficacy and safety of different plasma concentrations of pirfenidone in patients with IPF. The study included 99 patients with IPF orally treated with pirfenidone capsules for at least 52 weeks. Ultra-performance liquid chromatography–mass spectrometry was used to analyze drug plasma concentrations. The annual rate of forced vital capacity (FVC) decline, assessed at week 52, was set as the primary end point. Secondary end points were the change from the baseline in the 6-min walk distance (6 MWD) and the time to the first acute exacerbation of IPF, both of which evaluated over 52 weeks. In the total population, the annual FVC decline in the high-concentration group was −90.0 ml per year versus −260.0 ml per year in the low-concentration group, for a between-group difference of 190.3 ml per year. The proportion of patients treated with high plasma concentrations of pirfenidone who showed an absolute decline of ≥10% in FVC% predicted, with a 6 MWD reduction of ≥50 m, or died, was lower than that of patients treated with low plasma concentrations of pirfenidone. High concentrations of pirfenidone reduced the risk of acute exacerbation in patients with IPF. Considerable differences were not observed for the total St. George’s Respiratory Questionnaire score or the rates of death between the high- and low-concentration groups. Mild to moderate adverse events, mainly involving the gastrointestinal system and the skin, were more common in the high-concentration group than in the low-concentration group but did not lead to termination of treatment in most cases. Our results suggest that treatment of IPF with high blood concentration of pirfenidone is both safe and effective. In the case of tolerable adverse reactions, patients with IPF may benefit from high concentrations of pirfenidone.

## Introduction

Idiopathic pulmonary fibrosis (IPF) is a chronic and progressive fibrotic lung disease with usual interstitial pneumonia (UIP) as the main histopathological and radiological pattern. IPF causes honeycombing, irreversible tissue damage, and respiratory failure, which seriously affect the quality of life of patients and increase the mortality rate. IPF accounts for approximately 20% of all cases of interstitial lung diseases, with approximately 3 million IPF cases globally (Lederer and Martinez, 2018) and a high prevalence among the elderly and males. The survival time varies widely among patients with IPF (Raghu et al., 2011). Advanced age, male, dyspnea, the rapid decline of lung functions in the early stage of the disease (Raghu et al., 2020), and severe pathological injury are all associated with the poor prognosis of patients with IPF. Due to the lack of effective drug treatments, the median survival of transplant-free patients with IPF is 3–5 years, which is less than the survival rate of patients with other malignancies (Mora et al., 2017), with an extremely poor prognosis. Hence, the search for an effective therapeutic strategy for IPF is at the forefront of contemporary research (Collard et al., 2016; Kolb et al., 2017; Richeldi et al., 2017; Lederer and Martinez, 2018; Kondoh, 2019; Kirby, 2021).

The pathogenesis of IPF is extremely complex and hence can be easily misdiagnosed. Studies have shown that the epithelial-mesenchymal transition (Jayachandran et al., 2009; Wilson and Wynn, 2009), abnormal activation of lung fibroblasts (Zhao et al., 2016), and excessive deposition of collagen matrix (Martinez et al., 2017) play a key role in the process of pulmonary fibrosis.

Previous large-scale controlled clinical trial studies of IPF do not recommend the use of the combination of prednisone, azathioprine, and N- acetylcysteine as well as the administration of warfarin and endothelin receptor antagonists (Noth et al., 2012) due to their poor efficacy. The Food and Drug Association (FDA) has approved the antifibrotic drugs pirfenidone (Noble et al., 2011; King et al., 2014) and nintedanib (Richeldi et al., 2014; Bonella et al., 2015; Flaherty et al., 2019) for the treatment of IPF, as they suppress the decline in lung functions and delay disease progression. Pirfenidone, an oral pyridone derivative with anti-inflammatory, antioxidant, and antifibrotic properties (Lee et al., 1998; Schaefer et al., 2011), has been reported to regulate TGF-β expression in animal models of pulmonary fibrosis and can inhibit fibroblast and collagen synthesis (Schaefer et al., 2011).

A study showed that pirfenidone reduced the number of coughs per hour in patients with IPF by 35% and improved subjective cough indicators (van Manen et al., 2017). In addition, pirfenidone could significantly alleviate the deterioration of dyspnea by decreasing the change of FVC decline and the University of California and San Diego Shortness of Breath Questionnaire from baseline to 12 months in the patients with IPF treated with pirfenidone (Glassberg et al., 2019). In a prospective controlled trial, treatment with pirfenidone for 9 months improved vital capacity and significantly reduced the risk of acute exacerbation of IPF (Azuma et al., 2005). A retrospective analysis of ASCEND and CAPACITY004 and 006 studies reported that pirfenidone could reduce the incidence of disease progression events in patients with IPF (FVC% predicted decreased by more than 10%, 6 MWD decreased by more than 50 m, hospitalization and death) (Nathan et al., 2019).

In recent years, more patients with IPF are prescribed pirfenidone by professional respiratory physicians, and the recommended dose is 1,800 mg/d. However, pirfenidone causes side effects, mostly in the gastrointestinal tract (indigestion and loss of appetite) and skin (photosensitivity), and these adverse reactions worsen with dosage increase, resulting in poor compliance of patients. There are highly variable individual differences in adverse reactions, the dosage is not fixed, and there is a lack of a reliable blood concentration range of pirfenidone to monitor adverse reactions and clinical efficacy. In view of this, this study investigates the safety and efficacy of different plasma concentrations of pirfenidone in the treatment of patients with IPF and explores appropriate drug reference for clinical treatment decisions.

## Materials and methods

### Study design and patients

This study is a multicenter real-world study conducted in 16 centers in Henan Province. The study was approved by the Research and Clinical trial Ethics Committee of the First Affiliated Hospital of Zhengzhou University (Ethical Review No. 2020-KY-257).

The recruitment time was from July 2020 to August 2021, and the eligible patients were 40–85 years old. IPF was diagnosed based on the diagnostic guidelines for IPF by the ATS/ERS/JRS/ALAT in 2018 (Raghu et al., 2018); patients with image findings suggesting probable UIP were diagnosed by transbronchial lung biopsy or surgical lung biopsy. All the high-resolution computed tomographic images and lung histopathology specimens were reviewed uniformly by at least one expert chest radiologist and one pathologist. The range dose of pirfenidone was 1,200–1,800 mg/d, taken orally *ter in die* (t.i.d., i.e., three times a day). Other eligibility criteria were as follows: 1) An FVC % predicted of 50% or more. 2) A diffusion capacity of the lung for carbon monoxide (DLco) between 30 and 90% predicted. 3) A ratio of the forced expiratory volume in 1 s to the FVC that was equal to or greater than 0.80. 4) A 6-min walk distance (6 MWD) of over 150 m at baseline. Written informed consent was obtained from all patients.

Exclusion criteria were as follows: 1) Use of other antifibrotic drugs, including nintedanib, high-dose prednisone (>10 mg), immunosuppressants, rituximab, and N- acetylcysteine. 2) Participation in any study of IPF drugs during the preceding one month. 3) History of malignant tumors during the preceding 5 years. 4) Patients receiving antineoplastic therapy. 5) Patients suffering from active or latent tuberculosis during the preceding six months. If an acute exacerbation was reported at any time during the trial, the researcher had the choice to either start any other treatment or increase the dose as required.

### Treatment regimen

All patients had been treated with an oral pirfenidone capsule with a dose of ≥1,200 mg/d and a maximum dose of 1,800 mg/d. The drug was administered in three equal doses and taken with three meals. Physical examination, clinical laboratory tests, lung function test, 6 MWD test, and the analysis of total St George’s Respiratory Questionnaire (SGRQ) score were performed at baseline and at week 52. Telephone follow-up was carried out on the 2nd, 4th, 8th, 13th, 26th, 39th, and 52nd weeks to evaluate exercise tolerance and dyspnea, acute aggravation of symptoms, and hospitalization due to illness. Adverse drug reactions and treatment measures were documented meticulously. We assessed the adequacy and repeatability of all lung function results according to the American Thoracic Society standards (Culver et al., 2017). Safety outcomes were determined from clinical and laboratory evaluations and the records of adverse events that occurred within the 52 weeks and were coded using the Common Toxicity Criteria (CTC) version 2.0 of the National Cancer Institute.

### Measurement of pirfenidone plasma concentration

Serial blood samples were collected at the baseline. Peripheral venous blood samples (2 ml) were drawn from each patient prior to dosing and at 1.5, 2, 4, 6, and 8 h post-dosing. All blood samples were collected in EDTA tubes and centrifuged at × 3,500 g for 10 min to isolate the plasma. Ultra-performance liquid chromatography–tandem mass spectrometry (UPLC-MS/MS) was used to analyze the plasma concentrations of pirfenidone. The calibration curve covered the range of 0.2–20.0 mg/L. An analysis of quality control samples indicated good precision (coefficients of variation ≤4.2%) and accuracy (measured concentrations ≤4.7% from target concentrations) (Wang et al., 2021). The area under the curve (AUC) at baseline was evaluated using the linear trapezoidal method, whereas the AUC 8 h was multiplied by 3 to obtain the AUC 24 h.

### Outcomes

The primary endpoint was the annual rate of decline in the FVC over the 52-week period. According to the international guidelines (Culver et al., 2017), pulmonary function was measured at the baseline and at the 52nd week. The secondary end points were the assessment of absolute changes in the percentage of predicted FVC, DLco, 6 MWD reduction, and the time to the first confirmed or suspected acute exacerbation of IPF or death from baseline to week 52.

The implementation of the 6-min walk test was defined and controlled according to the standards defined and validated by du (Bois et al., 2011); additional control measures were taken according to the ERS/ATS guidelines for field walking testing (Holland et al., 2014). Disease progression was defined as a decline of greater than 10% in the absolute FVC % predicted, hospitalization due to respiratory diseases, or a drop of 50 m or more in the 6 MWD compared with the baseline measurement. Acute exacerbation of IPF was defined as the acute clinically significant deterioration of respiratory functions characterized by new evidence of extensive alveolar abnormalities, which met the following diagnostic criteria (Collard et al., 2016): 1) The previous or current diagnosis was IPF. 2) Acute deterioration or dyspnea, usually lasting <30 days. 3) Computed tomography showed bilateral ground glass shadows and/or overlapping consolidations of background types consistent with the common type of interstitial pneumonia. 4) A deterioration that does not account completely for heart failure or humoral overload. Cases that are of unknown cause but do not fulfill the criteria listed due to missing computed tomography data and have been termed “suspected acute exacerbations of IPF.” Additional secondary endpoints included the change in the total SGRQ score measured over the entire treatment period. The SGRQ, which is used to evaluate the quality of life, is a self-administered questionnaire that consists of three different domains (symptoms, activity, and impact). The score of each domain ranges from 0 to 100, and the total score represents the weighted average of the three sub-scores, with higher scores corresponding to a poorer quality of life (Jones et al., 1991; Furukawa et al., 2017; Prior et al., 2019).

### Statistical analysis

The research data were statistically analyzed using the SPSS25.0 software (IBM Corporation, Armonk, NY, United States). To analyze the mean change, the missing values due to death were assigned the worst result (e.g., FVC = 0). The normal distribution, skewed distribution, and counting data were expressed as average ±standard deviation, median (lower quartile, upper quartile), and frequency (constituent ratio), respectively. The T, χ2, and Mann-Whitney *U* tests were performed for comparisons among groups. The data were all double-tailed statistics, with an alpha value of 0.05. For time-to-event analyses, the high-concentration group was compared with the low-concentration group using the log-rank test.

## Results

### Patient characteristics

**FIGURE 1:**
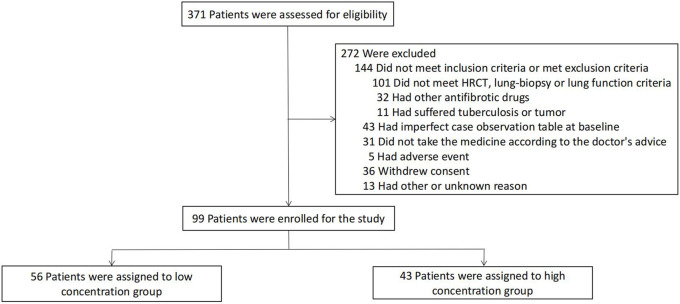
Enrollment and Grouped in the Overall Population. Patients could have more than one reason for exclusion. The numbers of patients who withdrew from the study do not include patients who died or underwent lung transplantation. Patients who discontinued the study treatment were included in the analysis of data for patients who completed the study.

From July 2020 to August 2021, 405 blood samples were collected from 371 patients treated with pirfenidone (≥1,200 mg/d), of which 31 collected two blood samples and 1 collected four blood samples while adjusting the drug dose. After excluding the patients with inadequate clinical features, test results, pulmonary functions, 6 MWD and SGRQ scores at baseline, and follow-ups within 52 weeks, along with the patients who could not be diagnosed as having IPF by imaging or pathology, 99 patients who completed the visits were finally enrolled in the study (Figure 1).

**FIGURE 2:**
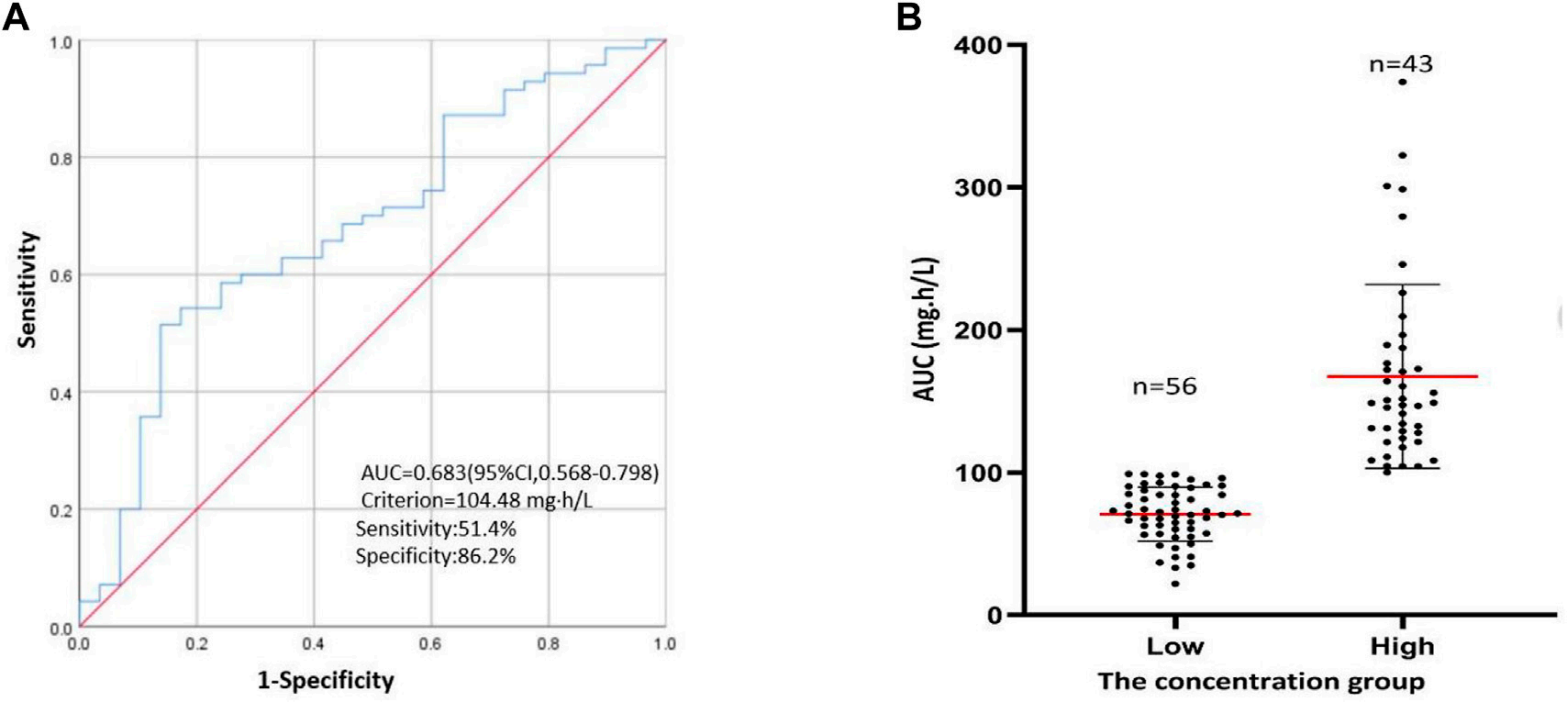
The concentration group. **(A)** ROC curve for values of pirfenidone plasma concentration to predict the progression in IPF patients. **(B)** The distribution for the two different concentrations, the horizontal red lines represent the mean value and the lower and upper black lines represent the S = D value, respectively.

A UPLC-MS/MS-based method for the simultaneous measurement of pirfenidone and its main metabolites in the plasma of the patients was established in our center. Blood samples were collected for drug concentration determination after repeated administration. The plasma concentration of pirfenidone in patients with IPF was 112.8 ± 65.5 mg h/L. Upon viewing the AUC of the receiver operating characteristic curve (Figure 2A), we found that pirfenidone exposure was strongly correlated with the progression of the disease, and the cut-off value was 104.483 mg h/L; the patients were assigned into two different plasma concentration groups: the low-concentration group (AUC lower than 100 mg h/L) and the high concentration group (AUC ≥100 mg h/L). The distribution of pirfenidone plasma concentrations showed considerable differences between the two groups (Figure 2B).

Overall, the two study groups were similar in terms of demographic and baseline characteristics (Table 1). Most enrolled patients in the low- and high-concentration groups were males (80.4%and 76.7%, respectively), with a median age and smoking history of 64 and 67 years and 66.1 and 58.1%, respectively. Most patients were diagnosed within the preceding 3 years, with 80.4% in the low-concentration group and 79.1% in the high-concentration group. The mean (±SD) baseline FVC of the predicted value was 78.6% ± 16.7% in the low-concentration group and 74.4% ± 18.5% in the high-concentration group. The baseline values of the 6 MWD were 452.5 (388.5–505.0) m in the low-concentration group and 415.0 (385.0–465.0) m in the high-concentration group; the percentage of predicted DLco was 53.6% ± 12.2% and 53.7% ± 14.9%, respectively.

**TABLE 1:** Characteristics of the patients at baseline.

Characteristic	Low concentration group (n = 56)	High concentration group (n = 43)	*p*-value
Male sex-- no. (%)	45 (80.4)	33 (76.7)	0.663
Age-- years	64.0 (60.8–70.0)	67.0 (64.0–73.0)	0.129
Height (cm)	166.5 ± 6.3	165.1 ± 8.1	0.289
Body weight (kg)	70.8 ± 11	68.7 ± 12.9	0.404
Smoking history-- no. (%)	37 (66.1)	25 (58.1)	0.419
Time since first diagnosis years-- no. (%)	56	43	0.987
<1	17 (30.4)	13 (30.2)	
1–3	28 (50.0)	21 (48.8)	
≥3	11 (19.6)	9 (20.9)	
Diagnostic mode-- no. (%)	56	43	0.469
HRCT	52 (92.9)	37 (86.0)	
transbronchial lung biopsy	2 (3.6)	2 (4.7)	
Surgical lung biopsy	2 (3.6)	4 (9.3)	
Hypertension disease-- no. (%)	16 (28.6)	13 (30.2)	0.857
Diabetes-- no. (%)	8 (14.3)	6 (14.0)	0.962
Coronary artery disease-- no. (%)	8 (14.3)	4 (9.3)	0.451
Chronic gastritis-- no. (%)	1 (1.8)	1 (2.3)	0.850
PPI-- no. (%)	5 (8.9)	6 (14.0)	0.430
Antihypertensive drug-- no. (%)	9 (16.1)	11 (25.6)	0.243
Hypoglycemic-- no. (%)	6 (10.7)	3 (7.0)	0.521
Antiplatelet medicines-- no. (%)	5 (8.9)	3 (7.0)	0.724
Lipid-lowering drugs-- no. (%)	7 (12.5)	3 (7.0)	0.366
Drugs for control hepatitis-- no. (%)	0 (0.0)	1 (2.3)	0.251
Glucocorticoid-- no. (%)	9 (16.1)	2 (4.7)	0.073
Use of supplemental oxygen-- no. (%)	18 (32.1)	13 (30.2)	0.839
Total SGRQ score	34.2 ± 14.0	36.8 ± 11.6	0.326
6 MWD(m)	452.5 (388.5–505.0)	415.0 (385.0–465.0)	0.128
FVC(L)	2.8 (2.0–3.3)	2.4 (1.8–3.2)	0.105
FVC%	78.6 ± 16.7	74.4 ± 18.5	0.229
DLco (mmol/min/kPa)	4.3 ± 1.0	4.1 ± 1.1	0.267
Dlco%	53.6 ± 12.2	53.7 ± 14.9	0.995

HRCT, High-Resolution Computed tomography; PPI, proton pump inhibitors; SGRQ, St George’s Respiratory Questionnaire. 6 MWD, 6-min walk distance; FVC, forced vital capacity. FVC %, the percentage of the predicted FVC. DLco, diffusion capacity of the lung for carbon monoxide. Dlco %, the percentage of the predicted DLco.

### Plasma concentration and clinical outcomes of treatment

**FIGURE 3:**
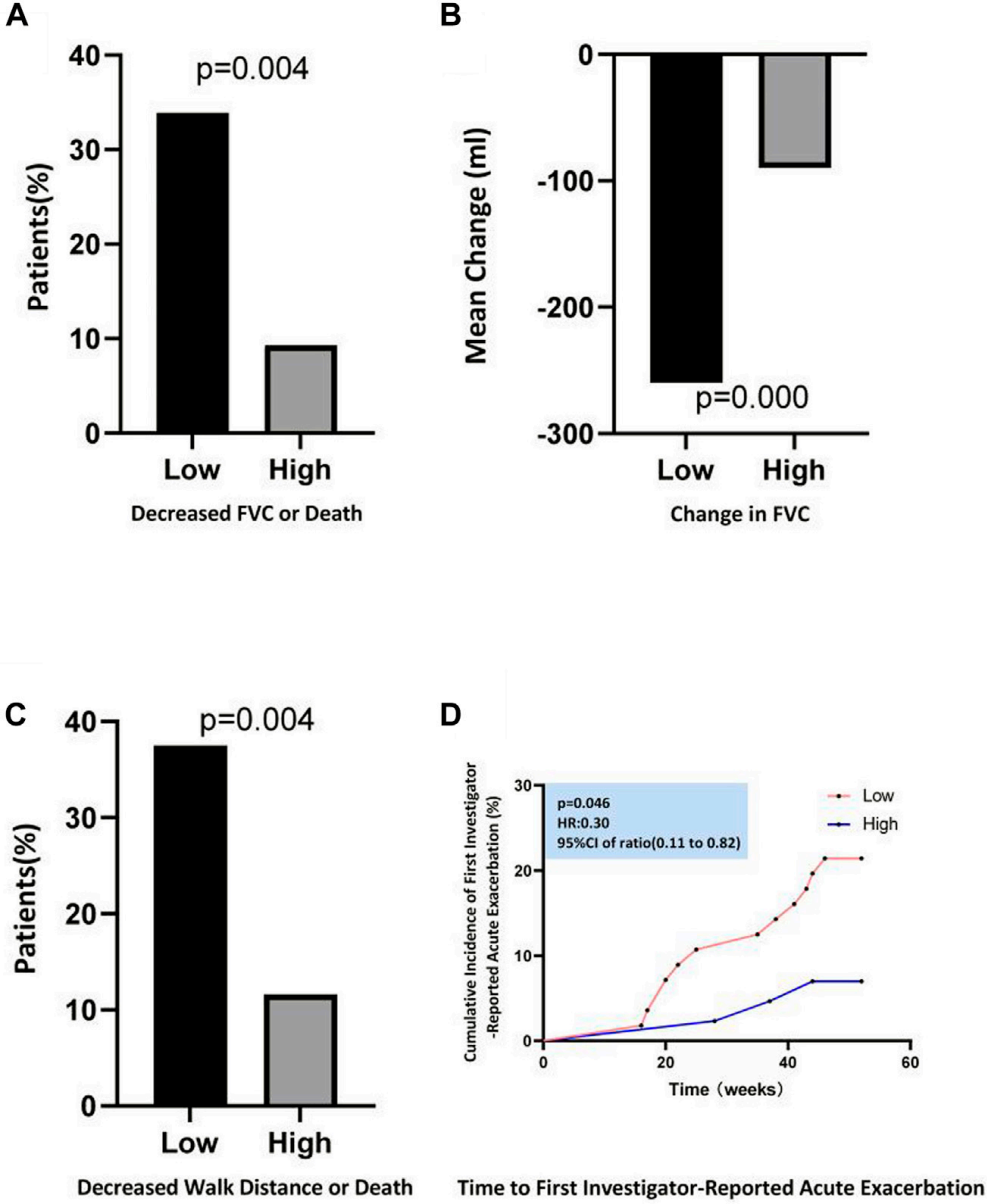
Efficacy Outcomes during the 52-Week Study Period. **(A)** Shows the proportion of patients who had a decreased percentage of the predicted FVC≥ 10% or who died. **(B)** Shows the mean change from baseline in FVC. **(C)** Shows the proportion of patients who had a decline of 6-min walk distance≥ 50 m or who died. **(D)** Shows the Kaplan–Meier distribution for the probability of time to first investigator-reported Acute Exacerbation.

After treatment with pirfenidone capsules, the adjusted annual rate of decline in the FVC (the primary end point) was −90.0 ml per year in the high-concentration group as compared with −260.0 ml per year in the low concentration group, with a difference of 190.3 ml per year (95% CI, 109.2–280.0; *p* < 0.001) (Table 2; Figure 3B).

**TABLE 2:** Efficacy endpoints.

End point	Low concentration group (n = 56)	High concentration group (n = 43)	Difference, high vs. low (95%CI)	*p*-value
ΔFVC(mL)	−260.0 (−390.0 to−120.0)	−90.0 (−190.0 to 80.0)	190.3 (109.2–280.0)	0.000
ΔFVC %	−7.6 (−11.4 to −3.0)	−2.5 (−5.4 to 3.0)	5.9 (3.2–8.6)	0.000
ΔDlco %	−7.9 (−12.8 to −4.1)	−5.3 (−9.0 to −2.0)	2.5 (−0.3–5.6)	0.078
Δ6MWD(m)	−40.0 (−70.0 to −15.0)	−15.0 (−40.0 to −5.0)	23 (5.0–35.0)	0.006
ΔTotal SGRQ score	2.9 (−1.8–5.6)	2.7 (0.8–4.4)	−0.3 (−2.1 to 1.6)	0.764

ΔFVC, the annual rate of decline in the FVC, from baseline to week 52. ΔFVC %, the absolute change in the percentage of the predicted FVC, from baseline to week 52. ΔDlco %, the absolute change in the percentage of the predicted Dlco from baseline to week 52. Δ6MWD, the absolute change in 6-min walk distance from baseline to week 52. ΔTotal SGRQ, score, the change from baseline to week 52 in the total score on the St. George’s Respiratory Questionnaire.

The FVC % predicted changed significantly during the 52-week treatment period between the two different plasma concentration groups (*p* < 0.001) (Table 2). The proportion of patients with a decline of ≥10% in the absolute FVC % predicted or who had died at week 52 was less in the high-concentration group than in the low concentration group (9.3% vs. 33.9%, *p* = 0.004) (Figure 3A). The high-concentration group showed a significantly lower decline over the 52-week period in the 6 MWD than the low concentration group (−15.0 m and −40.0 m, respectively, between-group difference, 23 m; 95% CI, 5.0–35.0; *p* = 0.006) (Table 2). At week 52, five patients (11.63%) in the high-concentration group and 21 patients (37.5%) in the low-concentration group had a 6 MWD reduction of more than 50 m or died, for a relative decrease of 28.6% in the high concentration group (Figure 3C).

During the assessment of changes in the percentage of predicted DLco from the baseline to week 52, the high- and low-concentration groups showed a decrease of −5.3 and −7.9% in DLco, respectively (between-group difference, 2.5%; 95% CI, −0.3 to 5.6; *p* = 0.078) (Table 2). At week 52, no significant difference in the average change in the total SGRQ score was observed between the two groups (2.7 and 2.9 in the high- and low-concentration groups, respectively; between-group difference, −0.3; 95% CI, −2.1–1.6; *p* = 0.764) (Table 2).

In the analyses of the time to the first acute exacerbation, a significant increase was observed in the high-concentration group compared with that in the low-concentration group (hazard ratio, 0.30; 95% CI, 0.11–0.82; *p* = 0.046) (Figure 3D); the percentage of patients with an acute exacerbation of IPF was lower in the high-concentration group than in the low-concentration group (7.0% vs. 21.4%) (Table 3). The all-cause mortality analysis showed two deaths (4.7%) over the 52-week period in the high-concentration group and three deaths (5.4%) in the low-concentration group; no significant difference was observed between the two groups (hazard ratio, 0.85; 95% CI, 0.14–5.09; *p* = 0.860) (Table 3). Similarly, the percentage of patients who died from IPF in the high-concentration group was lower than that in the low-concentration group, although the difference was not significant (*p* = 0.461) (Table 3).

**TABLE 3:** Outcomes of the trial.

Outcomes	Low concentration group (*n* = 56)	High concentration group (*n* = 43)	Hazard ratio (95% CI)	p-value
Acute exacerbation of IPF at Week 52-- no. (%)	12 (21.4)	3 (7.0)	0.30 (0.11–0.82)	0.046
Death from any cause-- no. (%)	3 (5.4)	2 (4.7)	0.85 (0.14–5.09)	0.860
Death related to IPF-- no. (%)	3 (5.4)	1 (2.3)	0.43 (0.04–4.10)	0.461

IPF, idiopathic pulmonary fibrosis.

### Plasma concentration and adverse events

Table 4 summarizes the adverse events associated with oral administration of pirfenidone in patients within the 52 weeks. In the overall population, the most common adverse events associated with oral pirfenidone were digestive tract-related adverse reactions such as nausea, vomiting, abdominal pain, diarrhea, dyspepsia, gastroesophageal reflux, weight loss, and rash. The total number of adverse events was 37 (86.0%) in the high-concentration group and 35 (62.5%) in the low-concentration group. There was a significant difference in the total incidence of adverse effects between the different concentration groups (*p* = 0.009).

**TABLE 4:** Adverse events in the overall population.

Event	Low concentration group (*n* = 56)	High concentration group (*n* = 43)	p-value
	no. of patients (%)		
Any	35 (62.5)	37 (86.0)	0.009
Nausea	20 (35.7)	27 (62.8)	0.007
Vomiting	5 (8.9)	9 (20.9)	0.089
Diarrhea, abdominal pain	2 (3.6)	6 (14.0)	0.060
Dyspepsia	19 (33.9)	24 (55.8)	0.029
Gastroesophageal reflux	23 (41.1)	20 (46.5)	0.588
Headache	4 (7.1)	7 (16.3)	0.152
Dizziness	2 (3.6)	3 (7.0)	0.443
Nasopharyngitis	0 (0.0)	2 (4.7)	0.103
Insomnia	5 (8.9)	8 (18.6)	0.158
Decrease in weight	14 (25.0)	19 (44.2)	0.045
arthritis	0 (0.0)	4 (9.3)	0.020
Rash	14 (25.0)	21 (48.8)	0.014
Allergy	5 (8.9)	7 (16.3)	0.267
Elevated bilirubin	1 (1.8)	0 (0.0)	0.378
Elevated transaminase	1 (1.8)	0 (0.0)	0.378
Elevated creatinine	1 (1.8)	0 (0.0)	0.378

In the high-concentration group, adverse events related to nausea, dyspepsia, weight loss, arthritis, and rash were more common compared with those in the low-concentration group. In the low-concentration group, one patient (1.8%) had grade 3 gastrointestinal adverse events, and two patients (3.6%) had grade 3 weight loss adverse events. One patient (2.3%) in the high-concentration group showed grade 4 skin-related adverse effects, while another (1.8%) in the low-concentration group showed grade 3 skin-related adverse effects. The adverse events were mostly mildly to moderately severe, easy to occur in the early stage of oral drug increment, and could be gradually tolerated or significantly relieved by drug treatment (such as proton pump inhibitors) or adjustment of living habits. In this study, liver-related adverse reactions were very rare. Only one patient (1.0%) had alanine or aspartate aminotransferase levels at least three times higher than the upper normal limit in the total population; the total bilirubin level of this patient increased to more than double the upper normal limit. However, the liver injury was reversible, and liver functions were restored after treatment with hepatoprotective drugs.

## Discussion

The recommended initial dosage for oral administration of pirfenidone capsules for patients with IPF is 200 mg thrice daily, and has been incrementally increased. The dosage of pirfenidone capsules is maintained at 600 mg per dose (1,800 mg daily). However, in clinical setting, patients with IPF are more susceptible to adverse events such as gastrointestinal reactions and skin rashes upon oral administration of 1,200 mg/day and these adverse reactions worsen as the dosage increase, and so does the cost of treatment, thereby causing financial burden on patients and affecting the incremental use of drug. Thus, it is pertinent to find an effective and safe blood concentration range of pirfenidone in patients with IPF. When we collated the data in the early stages, we found that ingesting higher doses of pirfenidone would increase the blood drug concentration in the same patient. However, no apparent correlation was observed between the dose and blood drug concentration in the overall population. Accordingly, it can be inferred that increasing the oral pirfenidone dose may increase the blood concentration of pirfenidone, resulting in a better therapeutic outcome.

In a multicenter clinical trial in Japan (Taniguchi et al., 2010), significant differences were observed in the vital capacity decline and progression-free survival (PFS) between the high-dose and placebo groups, but not between the high-dose (1,800 mg/d) and low-dose (1,200 mg/d) groups. The Ascend study (Richeldi et al., 2014) found that the annual FVC decline was 428 ml in the placebo group versus 235 ml in the pirfenidone group. At week 52, treatment with pirfenidone led to a markedly lower reduction in the 6 MWD and an improvement in the PFS. In this study, we found that the annual FVC decline (the main endpoint) was −90 ml in the high-concentration group and −260 ml in the low-concentration group. Additionally, the number of patients who showed a reduction of more than 10% in the absolute FVC % predicted, had a 6 MWD decrease of more than 50 m, or died at week 52 was considerably lower in the high-concentration group than in the low-concentration group. We found that the high plasma concentration of pirfenidone was beneficial in hindering the annual FVC decline and reducing the progression of the disease.

In previous clinical studies, it had been proved that pirfenidone had a positive effect on the decline of pulmonary function in the treatment of IPF, which not only reduced the annual decline rate of FVC but also slowed down the decline of DLCO (Feng et al., 2020; Krauss et al., 2020). In addition, DLCO decline of ≥10% shows potential as a mortality predictor in IPF patients on pirfenidone during follow-up examinations (Zurkova et al., 2019). Health-related quality of life (HRQL), often measured using the SGRQ is impaired in patients with IPF. This study showed no significant differences between the two different concentration groups regarding the changes in the percentage of predicted DLco (*p* = 0.078) or the total SGRQ score (*p* = 0.764) from the baseline to week 52. This may be because high-and low-concentration groups do not meet the differences in these indicators, and we need to enroll more patients to confirm these results.

Previous studies (Taniguchi et al., 2010; Noble et al., 2011; Richeldi et al., 2014) reported limited effects of pirfenidone on reducing the risk of acute exacerbation of IPF compared with that in the placebo group. However, the high plasma concentration of pirfenidone was beneficial in prolonging the time to the first acute exacerbation and reducing the risk of acute exacerbations, thus providing more evidence emphasizing the importance of monitoring the blood levels of pirfenidone.

A comprehensive analysis of data from three phase III clinical trials (Nathan et al., 2019) showed a significant reduction in the overall number of deaths (*p* = 0.01) and IPF-related deaths (*p* = 0.006) in the pirfenidone group compared with the placebo group. However, this study showed no significant differences between the two different concentration groups regarding the change in the overall deaths (*p* = 0.860), or deaths related to IPF (*p* = 0.461). This may be due to the low mortality rate in patients participating in IPF clinical trials, resulting in a small sample size not sufficient to acquire an accurate estimate of the therapeutic effect. Generally, pirfenidone capsule intake was safe. However, it was associated with some adverse events, the most common being digestive tract-related adverse events, weight loss, and rashes, which is consistent with the previous research findings (Wilson and Wynn, 2009; Raghu et al., 2011; Kolb et al., 2017; Martinez et al., 2017). The total incidence of adverse events in the high-concentration group (86.0%) was higher than that in the low-concentration group (62.5%). Adverse events (mostly mild to moderate) related to nausea, indigestion, weight loss, arthritis, and rash were more common in the high-concentration group than in the low-concentration group, but did not result in discontinuation of the drug.

This study has some limitations. 1) Its realistic design that did not alter the clinical treatment decisions of patients and the lack of a blank control group for comparison. 2) Relatively small sample size. 3) Patients with mild to moderate physical injuries were included. 4) The study groups were not divided by sex. IPF is a sex-dependent disease, and men and women could react to the treatment differently. 5) Some patients were on a long-term treatment of chronic diseases and had different lifestyles, which made it a tedious process to accurately evaluate the adverse reactions attributed to pirfenidone.

Despite these limitations, our multicenter study confirmed the advantages of the high plasma concentration of pirfenidone in the treatment of patients with IPF, such as delaying the annual decline rate of FVC, reducing the decline of 6 MWD, attenuating disease progression and the risk of acute exacerbation. Moreover, Treatment with pirfenidone capsules containing high plasma concentrations was generally safe, with tolerable side effects.

It is expected to carry out prospective researches and find a therapeutic window of the pirfenidone to monitor adverse reactions and clinical efficacy. It may achieve personalized medication of pirfenidone in the treatment of patients with IPF in the future.

## Data Availability

The raw data supporting the conclusion of this article will be made available by the authors, without undue reservation.
